# Supplementary material for: Longitudinal trajectories of sexual behavior and incident hepatitis C reinfection among men who have sex with men with HIV
Source: PLoS One. 2025 Jun 23;20(6):e0326094. doi: 10.1371/journal.pone.0326094 (PMC12184900; doi:10.1371/journal.pone.0326094)
Supplement: S1 Table — † Model 2 was chosen as the best fitting model based on lowest BIC and AIC. Model description: The best fitting model (2-class model) was based on the lowest BIC and AIC. Entropy is the degree of class separation ranging from 0–1, where an increased value indicates greater ability of the model to categorize persons into clusters. Abbreviations: AIC, Akaike information criterion; BIC, Bayesian information criterion; G, number of classes; NPM, number of estimated parameters. (DOCX) [file pone.0326094.s001.docx]

# **Supplementary Table 1.** Model fit statistics comparison for latent class analysis

|  | **G** | **NPM** | **Log-likelihood** | **BIC** | **AIC** | **Entropy** |
| --- | --- | --- | --- | --- | --- | --- |
| Model 1 | 1 | 12 | -1456.817 | 2971.381 | 2937.634 | 1 |
| Model 2^†^ | 2 | 22 | -1217.755 | 2541.378 | 2479.51 | 0.55 |
| Model 3 | 3 | 32 | -1408.207 | 2970.404 | 2880.414 | 0.58 |
| Model 4 | 4 | 42 | -1394.624 | 2991.360 | 2873.248 | 0.65 |
| Model 5 | 5 | 52 | -1389.791 | 3029.816 | 2883.583 | 0.66 |

^†^ Model 2 was chosen as the best fitting model based on lowest BIC and AIC.

**Model description**: The best fitting model (2-class model) was based on the lowest BIC and AIC. Entropy is the degree of class separation ranging from 0 – 1, where an increased value indicates greater ability of the model to categorize persons into clusters.

Abbreviations: AIC: Akaike Information Criterion; BIC: Bayesian Information Criterion; G: number of classes; NPM: number of estimated parameters
