# Supplementary material for: Longitudinal trajectories of sexual behavior and incident hepatitis C reinfection among men who have sex with men with HIV
Source: PLoS One. 2025 Jun 23;20(6):e0326094. doi: 10.1371/journal.pone.0326094 (PMC12184900; doi:10.1371/journal.pone.0326094)
Supplement: S2 Table — † Main Analysis. ‡ Sensitivity Analysis. § Posterior probabilities are not calculated for a 1-class model. From the latent profile analysis, an a posterior probability of an individual i belonging to each class is estimated using the maximum likelihood function (i.e., sums of the conditional likelihoods of each latent class, multiplied by the associated latent class probabilities). Mean posterior probabilities are calculated across individuals, indicating the average probability of their membership in the assigned latent class k. Higher mean posterior probabilities indicate a higher degree of confidence in the assigned class membership. Abbreviations: LCA, latent class analysis. (DOCX) [file pone.0326094.s002.docx]

# **Supplementary Table 2.** Mean posterior probabilities in each class per LCA model

|  | **Probability 1** | **Probability 2**^†^ | **Probability 3**^‡^ |
| --- | --- | --- | --- |
| **Model 1**^§^ | | | |
| **Model 2** | | | |
| **Class 1** | 0.84 | 0.16 | - |
| **Class 2** | 0.15 | 0.85 | - |
| **Model 3** | | | |
| **Class 1** | 0.80 | 0.10 | 0.10 |
| **Class 2** | 0.11 | 0.80 | 0.09 |
| **Class 3** | 0.03 | 0.15 | 0.82 |

^†^ Main Analysis.

^‡^ Sensitivity Analysis.

^§^ Posterior probabilities are not calculated for a 1-class model.

From the latent profile analysis, an *a* posterior probability of an individual *i* belonging to each class is estimated using the maximum likelihood function (i.e., sums of the conditional likelihoods of each latent class, multiplied by the associated latent class probabilities). Mean posterior probabilities are calculated across individuals, indicating the average probability of their membership in the assigned latent class *k*. Higher mean posterior probabilities indicate a higher degree of confidence in the assigned class membership.

Abbreviations: LCA: Latent class analysis
