# Supplementary figures and images for: Longitudinal trajectories of sexual behavior and incident hepatitis C reinfection among men who have sex with men with HIV
Source: PLoS One. 2025 Jun 23;20(6):e0326094. doi: 10.1371/journal.pone.0326094 (PMC12184900; doi:10.1371/journal.pone.0326094)

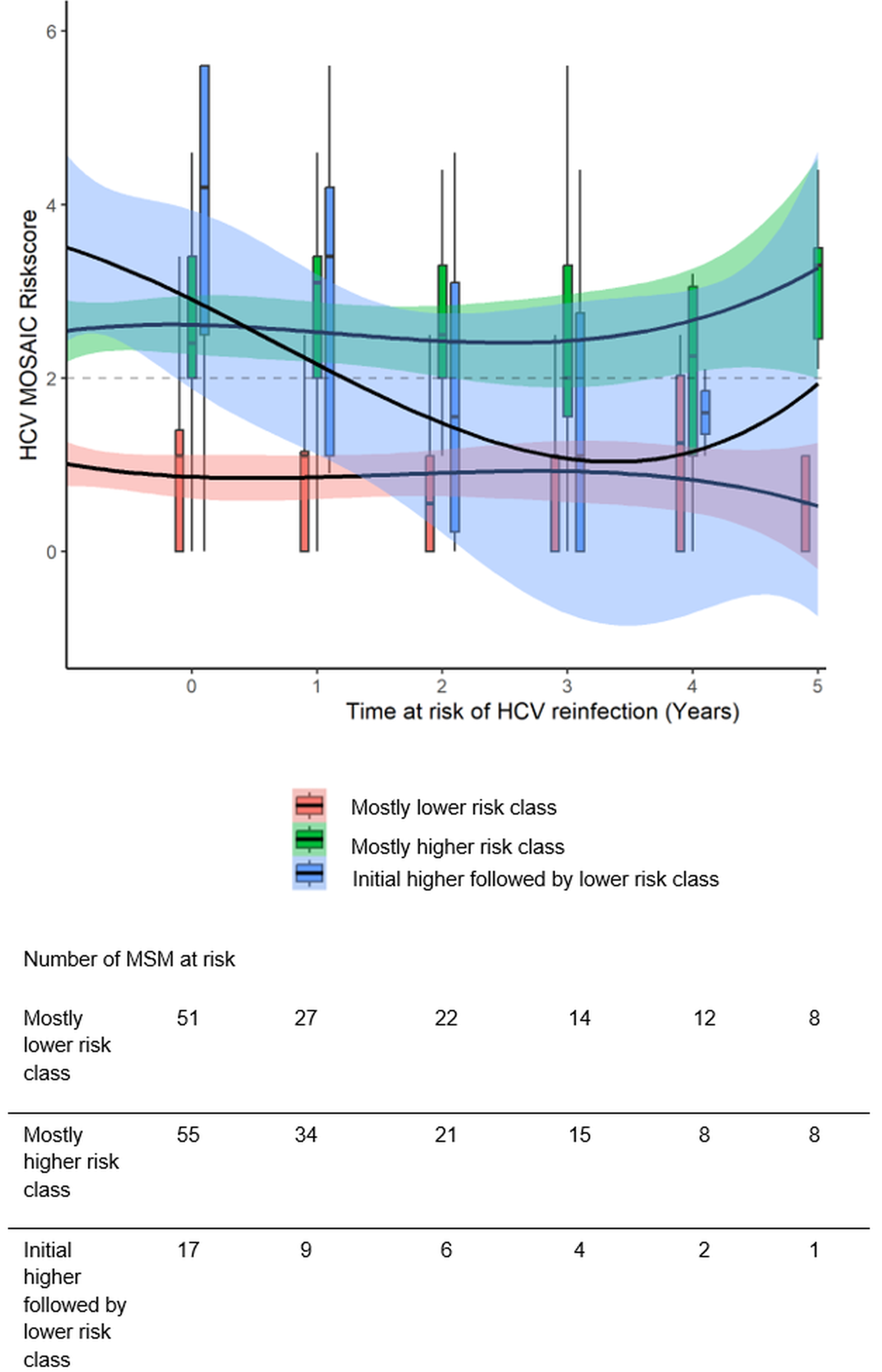

Supplement: S1 Fig — Boxes represent the median HCV-MOSAIC risk score and quartiles. The whiskers represent the minimum and maximum scores. The dashed line shows the validated HCV-MOSAIC cut-off ≥2 indicating higher risk for HCV [6]. The black solid lines represent the modeled HCV-MOSAIC risk score over time modeling using splines with 3-knots. The shaded areas represent the 95% CI. Individuals were assigned to the latent class (k) for which they had the highest posterior probability (πk), whereby πk was determined based on maximum likelihood. Abbreviations: CI, confidence interval; HCV, hepatitis C virus; MOSAIC, men who have sex with men Observational Study of Acute Infection with hepatitis C. (TIF) [file pone.0326094.s003.tif]

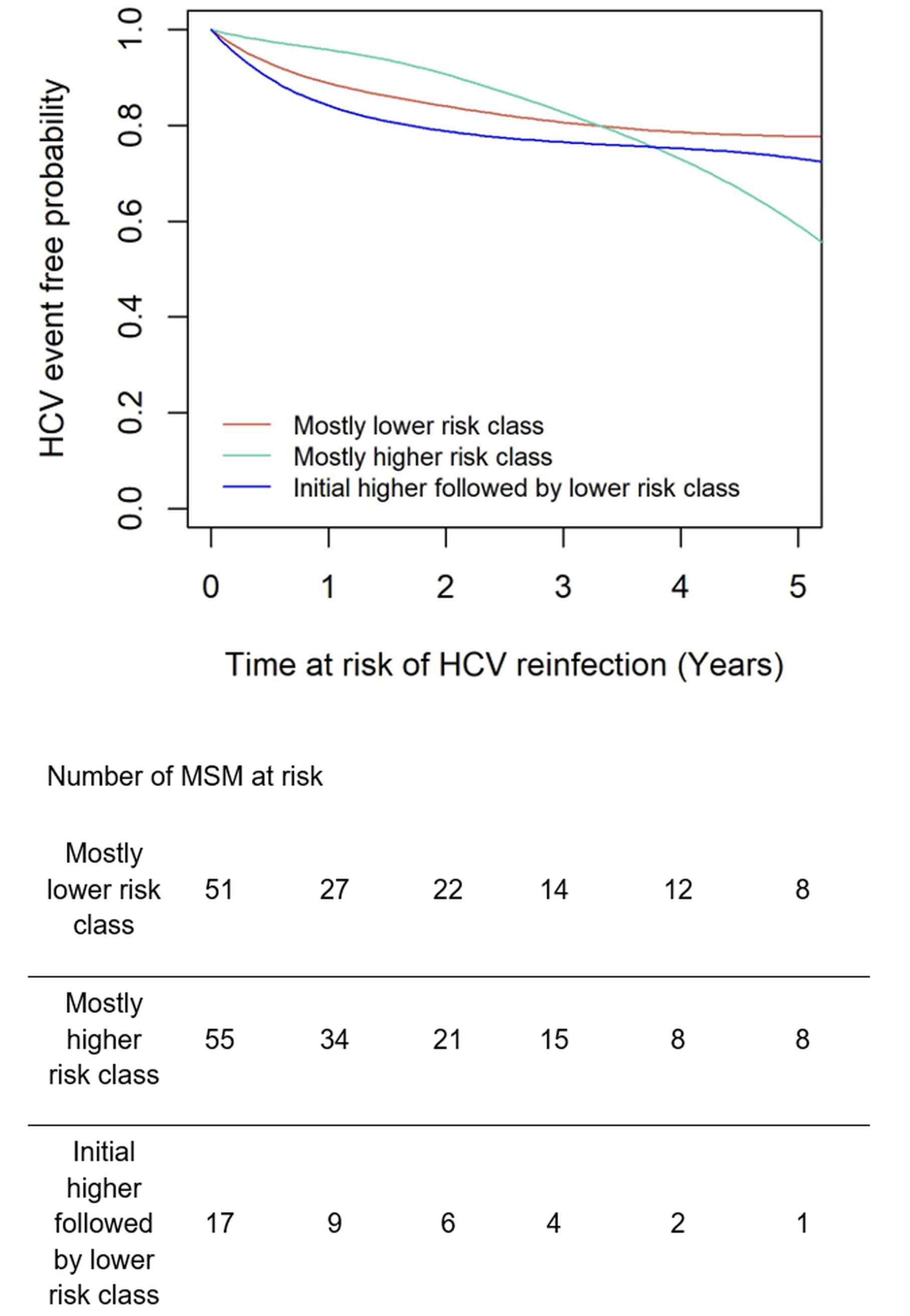

Supplement: S2 Fig — Fitted survival probabilities for remaining without HCV reinfection per assigned class from first visit since viral clearance to year 5 of follow-up. The model was fit using a joint survival model which combines the probability distributions from a linear mixed-effects model with random effects and a survival Cox model. Abbreviations: HCV, hepatitis C virus; MSM, men who have sex with men. (TIF) [file pone.0326094.s004.tif]
