# Supplementary material for: Longitudinal trajectories of sexual behavior and incident hepatitis C reinfection among men who have sex with men with HIV
Source: PLoS One. 2025 Jun 23;20(6):e0326094. doi: 10.1371/journal.pone.0326094 (PMC12184900; doi:10.1371/journal.pone.0326094)
Supplement: Attachment — Submitted filename: Response to Reviewers.docx [file pone.0326094.s006.docx]

Kris Hage

Nieuwe Achtergracht 100

1018 WT Amsterdam

The Netherlands

khage@ggd.amsterdam.nl

Amsterdam, 2 April, 2025

**To:** Dr. Syed Hani Abidi,

Academic Editor

**Re**: Revision of manuscript and author response to editor and reviewer comments

Dear Dr. Syed Hani Abidi,

We thank you for your consideration of our manuscript entitled “Longitudinal trajectories of sexual behavior and incident hepatitis C reinfection among men who have sex with men with HIV.” We wish to thank the Editorial team and reviewers for their valuable comments.

We have incorporated the Editorial and reviewers’ comments, as suggested. Please find our responses to each of these comments below. Changes are indicated in red font. Page numbers refer to the revised manuscript with tracked changes. As requested, we have also provided a version without marked changes.

We hereby also confirm that the work was supported by the “Aidsfonds” Netherlands (grant numbers 2008.026, 2013.037), the Netherlands Organization for Health Research and Development (ZonMw) (grant number 522004006) and GGD research funds. The funders had no role in study design, data collection and analysis, decision to publish, or preparation of the manuscript. Our competing interest disclosure does not alter our adherence to PLOS ONE policies on sharing data and materials.

We thank you for your considerations and hope that the revised version is suitable for publication in *PLOS ONE*.

Sincerely,

On behalf of all authors,

Kris Hage, MSc

**RESPONSES TO EDITORIAL COMMENTS**

| **Nr.** | **COMMENTS AND SUGGESTIONS FOR AUTHORS** | **RESPONSE AND REVISIONS** |
| --- | --- | --- |
| 1 | Please ensure that your manuscript meets PLOS ONE's style requirements, including those for file naming. | Thank you for your comment. The manuscript has now been formatted according to PLOS ONE’s style requirements, including file names. |
| 2 | Your abstract cannot contain citations. Please only include citations in the body text of the manuscript, and ensure that they remain in ascending numerical order on first mention. | We have carefully checked our abstract and made sure that citations are only included in the body text of the manuscript and not in the abstract. |
| 3 | Thank you for stating the following financial disclosure:  [This work was supported by the “Aidsfonds” Netherlands (grant numbers 2008.026, 2013.037), the Netherlands Organization for Health Research and Development (ZonMw) (grant number 522004006) and GGD research funds.].  Please state what role the funders took in the study. If the funders had no role, please state: ""The funders had no role in study design, data collection and analysis, decision to publish, or preparation of the manuscript.""  If this statement is not correct you must amend it as needed.  Please include this amended Role of Funder statement in your cover letter; we will change the online submission form on your behalf. | The funders indeed had no role in the study design, data collection and analysis, decision to publish, or preparation of the manuscript. The amended Role of Funder statement has been included in our Cover Letter above. The Cover Letter has also been uploaded as a separate file. |
| 4 | Thank you for stating the following in the Competing Interests section:  [I have read the journal's policy and the authors of this manuscript have the following competing interests: J. S.’s institution has received research support and consultancy fees from Gilead, and a speakers fee from Janssen Pharmaceuticals, independent from the submitted work. M. P.’s institution has received speakers fees and independent scientific support from Gilead Sciences, Roche, MSD, and Abbvie, outside the submitted work. M. V.’s institution has received consultancy fees from Gilead, MSD and ViiV outside the submitted work and research grants from Gilead, Merck Sharp Dome and ViiV, A.B. has received a speakers fee from Gilead Sciences, independent from the submitted work, grants from MSD and Gilead Sciences, and participated in advisory boards and received travel support from MSD, Janssen-Cilag, Bristol-Myers Squibb, Gilead Sciences, Pfizer, and ViiV Healthcare, outside the submitted work. All other authors report no potential conflicts. All authors have submitted the ICMJE Form for Disclosure of Potential Conflicts of Interest. Conflicts that the editors consider relevant to the content of the manuscript have been disclosed.].    Please confirm that this does not alter your adherence to all PLOS ONE policies on sharing data and materials, by including the following statement: "This does not alter our adherence to PLOS ONE policies on sharing data and materials.” (as detailed online in our guide for authors http://journals.plos.org/plosone/s/competing-interests).  If there are restrictions on sharing of data and/or materials, please state these. Please note that we cannot proceed with consideration of your article until this information has been declared.  Please include your updated Competing Interests statement in your cover letter; we will change the online submission form on your behalf. | Any of the information included in our conflict of interest disclosure does not alter our adherence to PLOS ONE policies on sharing data and materials. The updated Competing Interests statement has been included in our Cover Letter above. The Cover Letter has also been uploaded as a separate file.  The data used in this study are not publicly available due to the sensitivity of the information collected, in accordance with ethical and privacy regulations. The data are available upon reasonable request, subject to approval by the principal investigator. Please contact CoordinatieIZOZ@ggd.amsterdam.nl for more information and/or submission requests. |
| 5 | We note that you have indicated that there are restrictions to data sharing for this study. PLOS only allows data to be available upon request if there are legal or ethical restrictions on sharing data publicly. For more information on unacceptable data access restrictions, please see http://journals.plos.org/plosone/s/data-availability#loc-unacceptable-data-access-restrictions.  Before we proceed with your manuscript, please address the following prompts:  a) If there are ethical or legal restrictions on sharing a de-identified data set, please explain them in detail (e.g., data contain potentially identifying or sensitive patient information, data are owned by a third-party organization, etc.) and who has imposed them (e.g., a Research Ethics Committee or Institutional Review Board, etc.). Please also provide contact information for a data access committee, ethics committee, or other institutional body to which data requests may be sent.  b) If there are no restrictions, please upload the minimal anonymized data set necessary to replicate your study findings to a stable, public repository and provide us with the relevant URLs, DOIs, or accession numbers. For a list of recommended repositories, please see | The data used in this study are not publicly available due to the sensitivity of the information collected, in accordance with ethical and privacy regulations. The data are available upon reasonable request, subject to approval by the principal investigator. Please contact CoordinatieIZOZ@ggd.amsterdam.nl for more information and/or submission requests. |
| 6 | In the online submission form, you indicated that [Data are available upon reasonable request from the Principal Investigator (M. Prins, mprins@ggd.amsterdam.nl).].  All PLOS journals now require all data underlying the findings described in their manuscript to be freely available to other researchers, either 1. In a public repository, 2. Within the manuscript itself, or 3. Uploaded as supplementary information.  This policy applies to all data except where public deposition would breach compliance with the protocol approved by your research ethics board. If your data cannot be made publicly available for ethical or legal reasons (e.g., public availability would compromise patient privacy), please explain your reasons on resubmission and your exemption request will be escalated for approval. | Please refer to our answer to question 4 and 5 from the Editor. |
| 7 | Please review your reference list to ensure that it is complete and correct. If you have cited papers that have been retracted, please include the rationale for doing so in the manuscript text, or remove these references and replace them with relevant current references. Any changes to the reference list should be mentioned in the rebuttal letter that accompanies your revised manuscript. If you need to cite a retracted article, indicate the article’s retracted status in the References list and also include a citation and full reference for the retraction notice. | Thank you for your comment. We have carefully reviewed the reference list and verified that all citations are complete and correctly formatted. Please let us know if any further modifications are needed. |

**REVIEWER REMARKS**

| **Nr.** | **COMMENTS AND SUGGESTIONS FOR AUTHORS** | **RESPONSE AND REVISIONS** |
| --- | --- | --- |
| **Reviewer 1** | | |
| 1 | Even though this manuscript is quite interesting, this data is too old to be relevant at the current time. Also, a global pandemic happened, which impacted every aspect of life, including public health risks and interventions. From as old as 15 years and as early as six years is too long a time. | Thank you for your opinion and we understand the reviewer’s statement. First, we respectfully disagree with the reviewer that the global pandemic completely changed public health risks (and subsequent interventions) for HCV. Sexual behaviors in groups at risk of acquiring STI broadly had transient changes in sexual behaviors during the first lockdown [1,2]. From data collected in participants of a clinical trial who are at risk of HCV reinfection and among a nationwide prospective cohort of MSM with HIV, it became clear that COVID-19 pandemic had only a very temporary effect on behaviors associated with HCV and many are currently still engaging in at risk behaviors [3,4]. This elevated risk is corroborated with our national report on individuals living with HIV where we have still been observing 20-40 HCV re-infections per year in the years after the COVID-19 pandemic [5]. Second, the MOSAIC study is one of the few longitudinal cohort studies worldwide collecting detailed behavioral data related to HCV acquisition. These data allow us to explore trends and associations that could not be captured elsewhere. The basic demographic and sexual behavior characteristics from the data presented in this manuscript mirror those populations who are still at risk of HCV acquisition [6]. Despite the age of these data, we argue that they still have relevance to current populations. We have highlighted these limitations in the Discussion (page 20):  “Sixth, while we acknowledge the age of the presented data, we believe our findings remain valuable, given that COVID-19 pandemic-related changes in behaviors were transient and that our study population remain comparable to current populations at risk for an HCV infection [1,2,6].”  [1] Adam et al. 005.4 Changes in MSM’s sexual activity, PrEP use, and access to HIV/STI testing during and after the first Dutch COVID-19 lockdown. STI. 2021;97:A26.  [2] Jongen et al. Transient changes in preexposure prophylaxis use and daily sexual behavior after the implementation of COVID-19 restrictions among men who have sex with men. JAIDS. 2021;87(5):1111-18.  [3] Hage et al. The impact of the COVID-19 and mpox outbreaks on behaviours associated with HCV infection among MSM: preliminary results from the pre-randomization phase of a clinical trial. Poster 17^th^ Netherlands Conference on HIV Pathogenesis, Epidemiology, Prevention & Treatment 2024. Poster number P.23.  [4] Mugglin et al. Sexual behaviour and STI incidence in sexually active MSM living with HIV in times of COVID-19. Open Forum Infect Dis. 2023;10(8):ofad399.  [5] van Sighem et al. Monitoring Report 2024. Human Immunodeficiency Virus (HIV) Infection in the Netherlands. Amsterdam: Stichting hiv monitoring, 2024.  [6] Hage et al. Baseline analysis and cross-country differences among men who have sex with men enrolled in a randomized trial aimed at reducing risk of HCV reinfection: An update from the ICECREAM study. Abstract Fast-Track Cities 2024, Paris, France. |
| **Reviewer 2** | | |
| 1 | This is an interesting paper that using LCA has identified two classes of MSM with distinctive patterns of behavioral risk whose HCV reinfection risk diverged after 3 years of follow up. The main problem with this paper is what is the advantage for public health interventions of using the classification of MSM versus focusing on risk behaviors, given that the authors do not suggest targeted interventions but "frequent behavioral assessment is needed during care and should be extended over the years following HCV clearance". A further reflection on these implications is necessary for a recommendation for publications. | We thank the Reviewer for pointing out these shortcomings and agree that further reflection on the implications for public health interventions is needed.  There have been substantial declines in HCV incidence ever since the widespread use of DAAs. This might have led to overtesting key populations [1,2]. The goal of this analysis was to determine whether we could use longitudinal trajectories to differentiate those individuals at highest risk of HCV reinfection and hence, make HCV testing more efficient.  We were indeed able to differentiate these trajectories. However, considering that the level of risk behaviors tended to fluctuate over time, thereby making it difficult to predict behaviors in the future, we would have to rely on other characteristics to help identify these longitudinal clusters. Yet, there were no demographic or clinical characteristics that differentiated these groups, making it difficult to identify the trajectory group associated with higher rates of HCV acquisition at a single time point.  We conclude that trajectories of low and high levels of risk behavior with time exist, but given the difficulty in identifying at one time point who belongs in each, we can only recommend that assessment of HCV risk needs to be carried out continually. In addition, those suspected of being in the high level trajectory are likely to benefit from interventions on risk reduction interventions.  We have now included the following in the Discussion (page 21):  “These findings suggest that to inform when HCV testing is most needed, frequent behavioral assessment should be carried out over the years following HCV clearance. In addition, MSM who are suspected of belonging to the trajectory with consistently high levels of HCV-associated behaviors could be offered frequent testing and targeted behavioral interventions to prevent ongoing HCV transmission, as reductions in these behaviors are essential to curb the HCV epidemic in MSM [3].”  [1] Smit et al. HCV micro-elimination in individuals with HIV in the Netherlands 4 years after universal access to direct-acting antivirals: a retrospective cohort study. Lancet HIV. 2021;8(2):e96-e105.  [2] Sacks-Davis et al. Changes in incidence of hepatitis C virus reinfection and access to direct-acting antiviral therapies in people with HIV from six countries, 2010–19: an analysis of data from a consortium of prospective cohort studies. Lancet HIV. 2024;11:e106-16.  [3] Martin et al. Eliminating hepatitis c virus among human immunodeficiency virus-infected men who have sex with men in Berlin: A modeling analysis. J Infect Dis. 2019;220(10):1635-44. |
| **Reviewer 3** | | |
| 1 | **Abstract**  Here and elsewhere in the manuscript, where there are ranges of proportions presented, it would be more clear for the reader to include a % sign for both numbers in the range, as in “95%CI=11%-35%.” Consider spaces before and after the equal sign, as it seems there is enough room. | We thank the Reviewer for the reviewer’s comments and suggestions on the manuscript.  Agreed. We have now included spaces before and after the equal sign and included a % for both numbers in the range in the Abstracts (page 3):  “HCV reinfection probability was similar between both classes at year 3 of follow-up (C1, 17%, 95%CI = 11%-35% and C2, 18%, 95%CI = 15%-47%), but became higher in C2 than C1 at year 5 (C1, 22%, 95%CI = 13%-39% and C2, 37%, 95%CI = 28%-69%).”  These changes have also been included throughout the manuscript. |
| 2 | **Abstract**  In presenting the class 1 and class 2 results, please maintain the same order of the classes throughout, presenting first for C1 and then for C2. It would also be more clear to use consistent punctuation here and elsewhere in the manuscript, either “C1, result” or “C1: result.” Please be sure to add a space after the comma or colon. The numbers are very dense and needlessly run together. | Agreed. We have changed the following in the Abstract (page 3):  “HCV reinfection probability was similar between both classes at year 3 of follow-up (C1, 17%, 95%CI = 11%-35% and C2, 18%, 95%CI = 15%-47%), but became higher in C2 than C1 at year 5 (C1, 22%, 95%CI = 13%-39% and C2, 37%, 95%CI = 28%-69%). |
| 3 | **Introduction**  Lines 73-74. I would suggest “in 2014” since the verb is became and that happens only once. The alternative would be “have been widely available…since…” | Agreed. We have changed the following in the Introduction (page 4):  “Simplified therapy with direct-acting antivirals (DAAs), which provided higher cure rates and fewer side-effects compared to pegylated-interferon based therapy, became widely available in the Netherlands in 2014.” |
| 4 | **Introduction**  Lines 80-83. Important information is given about primary and reinfection rates that is left very vague, with no number presented. Please present numeric estimates of the primary and reinfection rates and the proportion of HCV incident cases from the reference cited. | Thank you for this suggestion. We have now included the following in the Introduction (page 4):  “While the incidence of primary HCV infection in the Netherlands has seen marked declines following broad access to DAAs [from 1.0/100 person-years (PY) in 2015 to 0.4/100 PYs in 2019], the incidence of HCV reinfection has also declined but remained relatively high at 1.1 /100 PYs in 2019 [1]. Accordingly, the proportion of HCV incident cases due to reinfection increased from 24% in 2015 to 35% in 2019 [2]. “  [1] Smit et al. HCV micro-elimination in individuals with HIV in the Netherlands 4 years after universal access to direct-acting antivirals: a retrospective cohort study. Lancet HIV. 2021;8(2):e96-e105.  [2] Sacks-Davis et al. Changes in incidence of hepatitis C virus reinfection and access to direct-acting antiviral therapies in people with HIV from six countries, 2010–19: an analysis of data from a consortium of prospective cohort studies. Lancet HIV. 2024;11:e106-16. |
| 5 | **Methods**  Lines 123-124. The question about gloves was apparently not very clear. If participants were asked “Were gloves used during fisting or were gloves shared?” then a yes response could either mean that gloves were used or that gloves were shared. Was there any way to tell if gloves were used but not shared? | Thank you for identifying these lacking details. To be clear, participants were asked whether fisting occurred with either steady or casual sex partners. If participants responded “yes”, they were asked whether gloves were used (yes/no) and when participants reported the use of gloves, they were additionally asked whether gloves were shared (yes/no). We have now included the following in the Methods (page 7):  “… (2) if fisting with sex partner(s) was reported, participants were first asked, “Were gloves used during fisting?”, and if the response was “yes”, participants were additionally asked, “Were gloves shared during fisting? ”,….” |
| 6 | **Methods**  Lines 161-162. “The incidence rate (IR) per 100 person-years (PY) of HCV reinfection and its corresponding 95% confidence interval (CI) was analyzed” – the verb should be “were analyzed” since there are two subjects. | Our apologies. It has now been changed to “were analyzed” in the Methods (page 8):  “The incidence rate (IR) per 100 PYs of HCV reinfection and its corresponding 95% confidence interval (CI) were analyzed by dividing the number of incident infections by the amount of person-time, overall and for each class.” |
| 7 | **Discussion**  Line 291. NAD has not been established as an abbreviation and is not used again. Please spell out. | Agreed. We have changed the following in the Discussion (page 18):  “In both classes, MSM demonstrated some variation in risk scores over time, which was mostly the result from changes in sharing sex toys, unprotected fisting, sharing straws when nasally administered drugs were used and receptive CAS.” |
| 8 | **Discussion**  Lines 300-301. Please add “an” before “individual’s sexual network.” | Agreed. This has now been changed in the Discussion (page 18):  “Changes in sexual relationships and being aware that HCV is circulating within an individual’s sexual network are important factors related to changes in sexual behaviors” |
| 9 | **Discussion**  Line 303. Please correct “prevention programs…was delivered.” | Agreed. We have changed the following in the Discussion (page 18):  “In addition, prevention programs with an emphasis on harm and risk reduction were delivered during the DAA era through the NoMoreC project.” |
| 10 | **Discussion**  Line 307. Please change “in demographic and clinical characteristics” to in demographic or clinical characteristics.” | Agreed. This has now been changed in the Discussion (page 18):  “There were no differences in demographic or clinical characteristics between classes at the first visit at risk for HCV reinfection.” |
| 11 | **Discussion**  Lines 311-312. Do the prior studies show younger age and lower CD4 counts to be potential determinants? Please be more specific about the direction of this association seen in other studies. | Thank you for this suggestion. Indeed, younger age and lower CD4 cell counts are considered potential determinants of HCV risk. We have included the following information to the Discussion (page 18):  “It is worth noting that prior research has suggested younger age and lower CD4+ cell counts as potential determinants of increased HCV risk, though the latter has yielded mixed results in various studies.” |
| 12 | **Discussion**  Limitations. It could be more clearly stated that the small sample size and relative low number of events were key limitations. For latent class analysis, 250-300 or more is often mentioned as a minimum sample size. | Thank you for this suggestion. Agreed. We have now included the following in the Discussion (page 20):  “Fifth, the few numbers of HCV reinfections produced fairly wide CIs for the IRs and survival probabilities, reducing power to effectively assess differences in HCV reinfection rates between classes.” |
| 13 | **Discussion**  Lines 354-356. Consider adding “in other settings or circumstances” to the end of this sentence. | Agreed. This has now been changed in the Discussion (page 20):  “Results might therefore not be generalizable to the wider MSM community with a resolved HCV infection or to settings where the epidemiological context of HCV is different.” |
| 14 | **Discussion**  Limitations. Reliance on self-reported behaviors and the potential for bias impacting results is not mentioned. | Thank you for this suggestion. We have now included the following to the Discussion (page 20):  “Furthermore, questions about engaging in sexual and drug use behaviors in the preceding months were self-reported and could therefore be biased by individual recall.” |
| 15 | **Discussion**  Limitations. Consider referring to PMID: 36398215 and discussing how different approaches to modeling risk over time can have important impact on results in latent class trajectory studies and therefore the results should be considered to be hypothesis-generating and should be confirmed in other studies. | Thank you for pointing out this useful methodological study. We have added the following to the Discussion (page 20):  “Although we used well established criteria to select the best-fitting model, these criteria could be influenced by model specification or even different functional forms of modeling time [1]. Future studies would be needed to validate these classes, while acknowledging that such data are rarely collected [2].”  [1] Watson et al. Latent class trajectory modelling: impact of changes in model specification. Am J Transl Res. 2022;14(10):7593-7606.  [2] Hage et al. Screening for hepatitis C virus reinfection using a behaviour-based risk score among men who have sex with men with HIV: Results from a case-control diagnostic validation study. Pathogens. 2023;12(10):1248. |
| 16 | Table 1. There is a footnote about p values and yet no p values are presented in this table. Please add these, as the comparisons are helpful and p values are included in the other comparison table (Table 2). | Thank you for identifying this mistake. We thank the Reviewer for pointing this out.  As the clinical characteristics and reported behaviors described in Table 1 are measured at different timepoint for both groups (at last study visit or at the moment of HCV reinfection), it is difficult to make a comparison. We have therefore decided to only describe characteristics without using a statistical test to compare groups. We have removed the footnote below Table 1. |
